# Maximum oxygen consumption and quantification of exercise intensity in untrained male Wistar rats

**DOI:** 10.1038/s41598-020-68455-8

**Published:** 2020-07-13

**Authors:** Fei Qin, Yanan Dong, Songtao Wang, Minxiao Xu, Zhongwei Wang, Chaoyi Qu, Yan Yang, Jiexiu Zhao

**Affiliations:** 1 China Institute of Sport Science, No. 11 Tiyuguan Road, Dongcheng District, Beijing, 100061 China; 2 School of Physical Education, Jinan University, Guangzhou, 510632 China; 3 School of Physical Education and Sport Science, South China Normal University, Guangzhou, 510006 China; 4 Department of Kinesiology and Community Health, University of Illinois at Urbana–Champaign, Champaign, 61801 USA; 5 Beijing Institute of Sports Science, Beijing, 100075 China

**Keywords:** Respiration, Lifestyle modification

## Abstract

This study aimed to explore a valid test protocol for measuring VO_2max_ in healthy untrained male Wistar rats of different ages and quantifying the exercise intensity (%VO_2max_) of running under different treadmill grades and speeds. The test protocols and %VO_2max_ will provide a reference for the design of exercise intensity. We tested male Wistar rats aged 4 weeks, 10 weeks, 10 months and 16 months old with three test protocols (Procedure 1 [P1], 2 [P2] and 3 [P3]) for each age group to quantify VO_2max_. We analysed VO_2max_, respiratory exchange ratio and test duration to determine an optimal test protocol of VO_2max_ for different age groups. We used the optimal test protocol to explore the changes in age-related VO_2max_. Finally, %VO_2max_ of running under different treadmill speeds and grades was quantified. VO_2max_ of Wistar rats decreased significantly after the age of 4 weeks (*p* < 0.05). The optimum VO_2max_ can be induced by personalised protocols for different ages. In 4-week-old Wistar rats, the highest VO_2max_ values were attained by P1 (104.4 ± 6.9 mL · kg^−1^ · min^−1^, *p* = 0.032). The highest VO_2max_ value (84.7 ± 3.7 mL · kg^−1^ · min^−1^, *p* = 0.037) of 8-week-old Wistar rats was attained in P2. In 10-month-old Wistar rats, the highest VO_2max_ value was obtained in P3 (63.3 ± 1.7 mL · kg^−1^ · min^−1^). This work could be used as a reference for assessing aerobic capacity in studies on exercise intervention with untrained male Wistar rats. However, the %VO_2max_ measurements at various treadmill speeds and grades only apply to untrained male Wistar rats.

## Introduction

Cardiorespiratory fitness (CRF) reflects the integrated ability to transport oxygen from the atmosphere to the mitochondria for performing large-muscle, dynamic, and moderate-to-vigorous intensity exercises for prolonged time periods. CRF is directly related to the integrated function of numerous systems, including the nervous, respiratory, cardiovascular and musculoskeletal systems^1–2^. CRF is considered a reflection of total body health. Low CRF is associated with a high risk of cardiovascular diseases, all-cause mortality and various types of cancer^3–5^. Hence, the American Heart Association (2016) regarded CRF as a new clinical vital sign with equal importance as the heart rate, blood pressure, respiration and body temperature^1^. CRF can be measured directly, expressed as maximal oxygen consumption (VO_2max_) or estimated from the peak work rate achieved on a treadmill, a cycle ergometer or through non-exercise algorithms^6^. VO_2max_ is the criterion measure of CRF^2^. In sports science, VO_2max_ has been used extensively to evaluate CRF and quantify exercise intensity for general populations and professional athletes^7,8^. Exercise intensity is a critical factor for people to achieve beneficial effects from physical activities. Such intensity is usually determined by VO_2max_ in the experimental design of sports science; thus, an objective and reliable test protocol should be formulated for VO_2max_ measurement^9^.

At present, VO_2max_ measurement and assessment have been well solved in human experiments and practices but are unsatisfactory in animal experiments. The accurate acquisition of VO_2max_ for experimental animals is also necessary for mechanism research in sports science. Bedford et al. developed and standardised the first test protocol of VO_2max_ for rats^10^. However, the features of experimental rats have changed largely over the past 40 years since the Bedford experiment. Therefore, quantifying exercise intensity on the basis of VO_2max_ data from previous studies may cause a deviation in calculation and a failure to obtain the expected experimental results. Additionally, some studies^11,12^ adopted too low or high exercise intensity, and such an approach often reduced exercise motivation and willingness and consequently affected the acquisition of the highest value of VO_2max_ in rats. To solve the above-mentioned problems, several crucial factors must be considered, namely, the differences in age, sex, strains, health status (health and disease) and training levels affecting the effectiveness of VO_2max_ test protocols^10,12,13^. For several special groups, a series of individualised VO_2max_ test protocols is needed. For instance, separate designs for VO_2max_ test protocols must be created for the oestrus cycle, perimenopausal period and menopausal period of female rats. Overall, effectively inducing the highest VO_2max_ value in experimental animals (rats) closely depends on the design of the test protocol, such as the intensity and duration of each exercise stage. Many clinical trials have implied that individualised test protocols of VO_2max_ should be utilised to obtain the largest VO_2max_ values for different populations. However, individualisation and differentiation for VO_2max_ tests in animal experiments according to age and health condition have yet to be conducted^10,13,14^. Hoydal reported that the working economy with low oxygen consumption improves during the training period^12^. Note that VO_2max_ will change in an exercise training program, and adjusting running speed according to VO_2max_ is necessary. Given the above factors, a series of work on individualised test protocols of VO_2max_ and a relative intensity calculation formula must be carried out in the future.

This study designed a series of standardised exercise protocols to measure VO_2max_ of male Wistar rats with the following objectives: (1) establishing a valid, reliable and individualised test protocol to measure VO_2max_ of male Wistar rats in different age groups; (2) exploring the relationship of VO_2max_ and age in male Wistar rats; and (3) quantifying the corresponding exercise intensity (%VO_2max_) of different treadmill grades and speeds in untrained male Wistar rats with different ages.

## Results

### VO_2max_ test protocol for male Wistar rats in different age groups

Table 1 shows the results of VO_2max_ indicators across the three procedures in 4-week-old Wistar rats. The induction rate of P1 achieved 75%, which was higher than those of P2 (63%) and P3 (63%). The VO_2max_ values in P1 (104.4 ± 6.9 mL · kg^−1^ · min^−1^ vs. 95.3 ± 6.9 mL · kg^−1^ · min^−1^, *p* = 0.032) and P2 (103.8 ± 8.9 mL · kg^−1^ · min^−1^ vs.95.3 ± 6.9 mL · kg^−1^ · min^−1^, *p* = 0.045) were obviously higher than those in P3. However, the completion time of P1 was significantly shorter than that of P2 (24.14 ± 2.63 min vs. 28.50 ± 2.81 min, *p* = 0.006). Unfortunately, four 4-week-old rats were injured in P1 and P2 because of poor adaptation to high treadmill speed.

**Table 1:** Related indicators of VO_2max_ in various test procedures for untrained male Wistar rats.

Parameters	P1	P2	P3
**4 weeks old**			
VO_2max_ (mL kg^−1^ min^−1^)	104.4 ± 6.9	103.8 ± 8.9	95.3 ± 6.9*^#^
RER	1.07 ± 0.24	0.98 ± 0.29*	1.04 ± 0.45^#^
Completion time (min)	24.14 ± 2.63	28.50 ± 2.81*	21.31 ± 2.5^#^
Induction rate	75%	63%	63%
Injured rats (n)	2	2	0
**8 weeks old**			
VO_2max_ (mL kg^−1^ min^−1^)	79.8 ± 4.4	84.7 ± 3.7*	80.3 ± 3.5
RER	1.04 ± 0.03	1.02 ± 0.03*	1.05 ± 0.02^#^
Completion time (min)	20.25 ± 3.83	24.00 ± 4.93	19.00 ± 2.59^#^
Induction rate	70%	75%	70%
Injured rats (n)	2	1	0
**10 months old**			
VO_2max_ (mL kg^−1^ min^−1^)	58.3 ± 2.2	62.2 ± 1.9	63.3 ± 1.7
RER	1.02 ± 0.02	1.03 ± 0.02	1.07 ± 0.01
Completion time (min)	14.50 ± 0.85	20.72 ± 1.43*	18.83 ± 1.38*
Induction rate	58%	75%	75%
Injured rats (n)	4	2	0

All values are presented as mean ± SD, *n* = 9 for each group.

*p < .0.05 versus P1; ^#^*p* < .0.05 versus P2. P1: Procedure 1, P2: Procedure 2 and P3: Procedure 3. Induction rate: a ratio of VO_2max_ induced cases to total tested rats’ cases.

As shown in Table 1, the highest VO_2max_ value (84.7 ± 3.7 mL · kg^−1^ · min^−1^, *p* = 0.037) of 8-week-old Wistar rats was attained in P2. However, P2 consumed more time than P3 did (24.00 ± 4.93 min vs. 19.00 ± 2.59 min, *p* = 0.021). In addition, two rats were injured in P1, and one was injured in P2.

Regarding the 10-month-old Wistar rats, the highest VO_2max_ value was found in P3 (Table 1). The test duration of P1 was remarkably shorter than those of P2 (14.50 ± 0.85 min vs. 20.72 ± 1.43 min, *p* = 0.002) and P3 (14.50 ± 0.85 min vs. 18.83 ± 1.38 min, *p* = 0.001). However, four rats were injured in P1. The rats showed lower compliance in P1 compared with the other procedures. Therefore, the induction rate of VO_2max_ in P1 was relatively low (58%).

### Relationship of VO_2max_ and ages in male Wistar rats

VO_2max_ of 8-week-old rats declined significantly compared with that of 4-week-old rats (105.6 ± 4.8 mL · kg^−1^ · min^−1^ vs 85.4 ± 4.0 mL · kg^−1^ · min^−1^, *p* < 0.05, Table 2). In addition, VO_2max_ of 10-month-old rats presented a downward trend compared with that of 8-week-old rats (85.4 ± 4.0 mL · kg^−1^ · min^−1^ vs. 62.3 ± 6.4 mL · kg^−1^ · min^−1^, *p* < 0.05, Table 2). Compared with 10-month-old rats, 16-month-old rats exhibited a 9.8% decline of VO_2max_ (62.3 ± 6.4 mL · kg^−1^ · min^−1^ vs. 56.2 ± 2.5 mL · kg^−1^ · min^−1^, *p* < 0.05, Table 2).

**Table 2:** Influence of age on related indicators of VO_2max_ in untrained male Wistar rats.

Age	VO_2max_ (mL kg^−1^ min^−1^)	RER	Completion time (min)
4 weeks	105.6 ± 4.8	1.035 ± 0.066	25.08 ± 3.69
8 weeks	85.4 ± 4.0*	1.034 ± 0.544	21.73 ± 3.66*
10 months	62.3 ± 6.4*^#^	1.044 ± 0.043	16.71 ± 2.67*^#^
16 months	56.2 ± 2.5*^#&^	1.071 ± 0.039*^#^	13.70 ± 2.23*^#&^

All values are presented as mean ± SD, *n* = 20 for each group.

*p < .0.05 versus 4-week-old rats. ^#^*p*  < .0.05 versus 8-week-old rats. ^&^*p*  <  0.05 versus 10-month-old rats.

### Quantification of the corresponding exercise intensity (%VO_2max_)

Tables 3, 4, 5 show the relative exercise intensity (%VO_2max_) in different grades (0°, 5°, 10° and 15°) and speeds (increased by 5 or 3 m min^−1^ every 3 min) among 4-week-old, 8-week-old and 16-month-old Wistar rats. We divided the actual measured value of VO_2_ when the rats were running at different speeds and grades by VO_2max_ to obtain the corresponding %VO_2max_ intensity. For example, at a treadmill speed of 10 m min^−1^ and slope of 0°, VO_2_ of 8-week-old Wistar rats was 41.1 ± 2.0 mL kg^−1^ min^−1^ and equivalent to 48.49 ± 2.79% VO_2max_. When the speed was 45–50 m min^−1^, the relative workload was 95%–100% VO_2max_ (Table 5). The data in Tables 3, 4, 5 can be used to estimate the exercise intensity at baseline for male Wistar rats at different ages. Note that these data only apply to untrained rats, and that the relative exercise intensity (%VO_2max_) cannot be used as a reference for trained rats.

**Table 3:** % VO_2max_ in different treadmill speeds and grades for 4-week-old untrained male Wistar rats.

Grade (°)	Speed (m min^−1^)										
	0	10	15	20	25	30	35	40	45	50	55
**0**											
VO_2_ (mL kg^−1^ min^−1^)	39.6 ± 4.7	55.5 ± 7.7	59.8 ± 7.9	61.3 ± 7.9	64.5 ± 6.6	70.6 ± 5.4	78.1 ± 5.0	85.8 ± 4.4	92.6 ± 6.5	101.6 ± 4.3	108.0 ± 6.9
%VO_2max_	35.79 ± 4.63	52.05 ± 4.59	59.05 ± 5.78	60.84 ± 5.56	64.25 ± 6.21	70.11 ± 5.80	78.67 ± 8.91	86.65 ± 7.47	93.28 ± 6.50	95.59 ± 2.88	100
**5**											
VO_2_ (mL kg^−1^ min^−1^)	38.9 ± 5.5	55.9 ± 4.6	60.7 ± 4.1	65.5 ± 4.6	70.1 ± 6.3	76.3 ± 7.3	82.8 ± 6.8	91.2 ± 7.7	99.5 ± 7.0	106.2 ± 4.4	108.7 ± 4.0
%VO_2max_	34.07 ± 0.80	52.26 ± 8.57	58.39 ± 7.91	63.13 ± 8.10	67.83 ± 9.45	73.96 ± 11.27	79.96 ± 9.37	88.03 ± 8.91	95.86 ± 6.70	97.34 ± 4.03	100
**10**											
VO_2_ (mL kg^−1^ min^−1^)	38.7 ± 3.3	55.3 ± 6.8	60.9 ± 9.4	69.1 ± 6.0	77.2 ± 10.6	85.1 ± 10.0	95.8 ± 6.8	98.9 ± 6.8	101.3 ± 6.4		
%VO_2max_	34.95 ± 4.35	52.18 ± 5.36	58.59 ± 8.49	65.72 ± 7.19	73.57 ± 11.29	81.30 ± 12.04	91.03 ± 7.86	94.59 ± 7.53	96.68 ± 8.33		
**15**											
VO_2_ (mL kg^−1^ min^−1^)	37.9 ± 2.7	56.0 ± 5.0	65.4 ± 5.8	76.5 ± 6.4	85.0 ± 4.7	89.4 ± 5.0	93.8 ± 4.6	98.0 ± 5.0			
%VO_2max_	34.22 ± 4.21	53.70 ± 7.63	62.76 ± 9.80	72.55 ± 10.15	80.36 ± 7.16	85.78 ± 4.85	89.46 ± 3.15	93.26 ± 4.41			

All values are presented as mean ± SD, *n* = 10 for each group. %VO_2max_ is the ratio of VO_2_ to VO_2max_.

**Table 4:** %VO_2max_ in different treadmill speeds and grades for 8-week-old untrained male Wistar rats.

Grade (°)	Speed (m min^−1^)									
	0	10	15	20	25	30	35	40	45	50
**0**										
VO_2_ (mL kg^−1^ min^−1^)	27.2 ± 4.5	41.1 ± 2.0	48.9 ± 5.0	54.3 ± 8.0	58.8 ± 9.1	61.2 ± 6.7	68.3 ± 9.0	73.8 ± 5.9	80.6 ± 1.5	87.1 ± 2.9
%VO_2max_	34.62 ± 6.80	48.49 ± 2.79	56.38 ± 4.82	61.30 ± 6.39	66.98 ± 9.83	72.64 ± 9.73	79.64 ± 10.07	89.81 ± 6.27	95.96 ± 4.57	100
**5**										
VO_2_ (mL kg^−1^ min^−1^)	30.3 ± 5.8	43.7 ± 5.4	49.0 ± 7.3	54.7 ± 9.4	60.7 ± 11.1	70.2 ± 9.9	78.4 ± 8.0	79.0 ± 5.8	89.0 ± 4.0	
%VO_2max_	34.90 ± 5.62	53.35 ± 4.52	59.72 ± 5.73	66.53 ± 6.33	73.80 ± 7.46	85.68 ± 7.21	91.95 ± 5.53	92.19 ± 2.80	100	
**10**										
VO_2_ (mL kg^−1^ min^−1^)	28.6 ± 5.6	44.0 ± 5.8	50.8 ± 6.9	59.2 ± 8.5	67.4 ± 9.2	76.5 ± 3.8	84.7 ± 5.6	90.3 ± 5.5		
%VO_2max_	33.25 ± 5.21	51.37 ± 5.20	59.51 ± 8.74	69.65 ± 12.55	81.72 ± 11.70	89.89 ± 9.09	97.60 ± 3.04	100		
**15**										
VO_2_ (mL kg^−1^ min^−1^)	28.3 ± 2.4	44.2 ± 2.9	54.4 ± 6.7	63.3 ± 9.8	66.8 ± 7.8	68.9 ± 0.3	74.0 ± 3.4			
%VO_2max_	33.88 ± 5.72	52.66 ± 3.26	63.93 ± 5.84	74.51 ± 8.41	84.44 ± 6.47	85.57 ± 4.08	90.43 ± 5.21			

All values are presented as mean ± SD, *n* = 10 for each group. %VO_2max_ is the ratio of VO_2_ to VO_2max_.

**Table 5:** % VO_2max_ in different treadmill speeds and grades for 16-month-old untrained male Wistar rats.

Grade (°)	Speed (m min^−1^)						
	0	12	15	18	21	24	27
**0**							
VO_2_ (mL kg^−1^ min^−1^)	19.3 ± 3.4	31.5 ± 4.4	40.9 ± 4.5	47.7 ± 5.5	51.3 ± 5.9	54.1 ± 4.8	54.7 ± 3.3
%VO_2max_	35.04 ± 6.68	57.04 ± 6.89	74.48 ± 9.51	86.62 ± 11.40	92.95 ± 10.07	98.31 ± 2.21	100
**5**							
VO_2_ (mL kg^−1^ min^−1^)	19.0 ± 2.5	32.4 ± 3.5	41.1 ± 5.7	47.4 ± 5.3	51.7 ± 4.8	52.9 ± 5.3	
%VO_2max_	35.23 ± 8.99	61.77 ± 6.35	78.16 ± 9.13	90.28 ± 7.73	98.72 ± 2.27	100	
**10**							
VO_2_ (mL kg^−1^ min^−1^)	17.8 ± 2.7	31.4 ± 3.2	39.8 ± 3.6	48.2 ± 5.1	52.1 ± 5.4		
%VO_2max_	34.53 ± 7.02	60.42 ± 5.81	76.76 ± 7.39	92.46 ± 2.16	100		

All values are presented as mean ± SD, *n* = 10 for each group. %VO_2max_ is the ratio of VO_2_ to VO_2max_.

## Discussion

In this study, we designed three test protocols to measure VO_2max_. Preliminary experiments indicated that the treadmill speed of less than 9 m min^−1^ easily results in backward running and reduced exercise motivation in rats. In accordance with the test protocols by Hoydal et al. and Wisloff et al.^12,13^, the initial speed should be more than 9 m min^−1^. In accordance with human studies, stable oxygen uptake could be achieved after 3–4 min in each exercise stage^15^. Similarly, stable oxygen uptake could usually be achieved within 3 min in animal tests. Therefore, we set the duration of each exercise stage as 3 min, which was consistent with the reports of Chavanelle et al. and Bedford et al.^10,16^. Moreover, several young rats were prone to demonstrate a sham plateau response that may be related to the duration of each exercise stage^17^. Furthermore, age was proportional to the duration needed for stabilising oxygen uptake in each exercise stage. Thus, the duration of each exercise stage should be adjusted in accordance with the subjects’ age. If the duration is unreasonably long, then the oxygen consumption would not increase continuously, thereby underestimating the VO_2_ peak and reducing running economy^12,17^. Regarding the termination criteria of the VO_2max_ test, Taylor et al. and Taylor et al. used the VO_2max_ plateau response in their studies, that is, an under 5% increase in VO_2_ with an increase in work intensity^18,19^. However, no significant statistical differences were found between plateau and non-plateau results in the same animals^10^. Consequently, the respiratory exchange ratio (RER), exhaustion state and blood lactate acid should be considered in addition to the volume change of O_2_ consumption when evaluating the standard of VO_2max_.

In the prophase of this study, we attempted to use Bedford et al.’s protocol. However, the protocol resulted in a low measured value of VO_2max_ and a high injury rate probably because of the overly high treadmill speed in each stage^10^. Thus, we appropriately reduced the increasing amplitude of speed and slope and named this procedure P1. As shown in Table 2, the highest VO_2max_ value of 75% in 4-week-old Wistar rats was induced by P1. Remarkably, the induction rate of VO_2max_ in P1 declined with the increase in age of Wistar rats (Table 1). During testing in P1, the older rats showed more serious stress response, poorer compliance and a higher injury rate (four injured rats) compared with the young rats. Meanwhile, few older rats reached the exhaustion state, and a low induction rate of VO_2max_ (58%) was obtained. Overall, P1 was more suitable for young rats than old rats, and the actual VO_2max_ value could be easily obtained. With regard to the incremental speed protocol (P2), the completion duration was the longest in the three procedures for all ages of rats. This result may be related to the single changing parameter, that is, treadmill speed. Therefore, an unreasonably low or high speed affected the final test results of VO_2max_. On this basis, the incremental speed of each stage in P2 should be adjusted in accordance with the rats’ age, physical condition and exercise capacity.

Compared with the young rats, older ones suffered more stress as speed increased. Hence, extremely high speed often reduced motivation and willingness for treadmill running. The rapid increase in speed in a short time could not fully mobilise the cardiorespiratory function in rats, thereby affecting the stress response. Thus, in P3, the treadmill speed was gradually increased until 80–85% of VO_2max_ intensity was reached. This speed was maintained, and the slope was increased for each stage. In this manner (P3), older rats still showed higher compliance than young ones during the latest age of the test, thereby presenting an ideal VO_2max_ value. Similar results were also found by Wisloff et al. and Fitzsimons et al.^13,20^. This similarity may be related to the increased treadmill slope that could enable considerably large muscles to work under a relatively low running speed, thereby reducing the stress response in rats^13,20^. Meanwhile, the risk of injury obviously declined in P3 for the older rats. The 4- and 8-week-old rats finished the VO_2max_ test in P3, and all rats were exhausted. Moreover, the highest VO_2max_ value in P3 was difficult to obtain in comparison with those in P1 and P2. This difficulty was possibly due to the progressive increasing slope that could not fully activate the cardiopulmonary function of young rats to the maximum level before exhaustion^16^. Thus, when measuring VO_2max_, slope-based programs (P3) should be given to older rats, whereas speed-based programs (P1 and P2) should be applied for young rats.

In the second stage of this study, we selected optimum test protocols obtained from the preceding research to quantify VO_2max_ of Wistar rats with different ages. As shown in Table 2, VO_2max_ in Wistar rats declined with age. This result was similar to those of Mazzeo et al. and Lawler et al.^14,21^. Our findings demonstrated the changing trend of VO_2max_ with age and provided a reference value of VO_2max_ in experimental rats for further studies.

A dose–response relationship must be observed strictly between exercise intensity/load and intervention effect in sports training and exercise prescription. Accurate exercise intensity (%VO_2max_) may only be obtained from directly measuring VO_2max_. Most studies failed to measure VO_2max_ because of lack of necessary equipment. Therefore, exercise intensity by %VO_2max_, especially in animal experiments, could not be quantified accurately. Nonetheless, in this work, the relative exercise intensities at different treadmill grades and speeds were quantified by calculating %VO_2max_ for the 4-week-old, 8-week-old and 16-month-old male Wistar rats. Hence, this study (Tables 3, 4, 5) could provide a reference for the initial design of exercise intensity in the exercise intervention research on untrained male Wistar rats. The main reason for the above limitations was the improvement in working economy with low oxygen consumption during the training period, thereby shifting the relationship between %VO_2max_ and treadmill grades and speeds significantly^12,13^. Note that the relative training intensities (%VO_2max_, Tables 3, 4, 5) were only applied to untrained rats or subjects for the initial design intensity. Hoydal indicated that the control of exercise intensity and the integrated effects of training require regulation of running speed according to the serial measurements of VO_2max_^12^.

## Limitations

Several limitations of this study should be noted. Firstly, differences in age, sex, strains and health status affected the test effects of VO_2max_; thus, a series of individualised protocols is needed. The VO_2max_ relative tests of male Wistar rats were part of our projects. In the future, the VO_2max_ tests of male Sprague–Dawley (SD) rats, the individualised protocol for designing different physiological states of female rats (the oestrus cycle, perimenopausal period and menopausal period) and the changes in VO_2max_ in several abnormal health statuses (obesity and chronic metabolic diseases) need further investigation. Secondly, the relative training intensity (%VO_2max_) was only applied to untrained rats or subjects for the initial design intensity. The indirect formula of VO_2max_ of rats during exercise training still needs more research support. Thirdly, in establishing the VO_2max_ test protocol for aging rats, we did not select 16-month-old rats due to poor exercise performance and serious casualties. One 16-month-old rat could not complete all three protocols, so the comparison could not be carried out. Therefore, we selected younger old rats (10 months old) to finish the establishment and modification of the protocol. Lastly, a sham plateau occurred in the VO_2max_ test, especially for the young rats. This occurrence may be related to the exercise intensity and the duration of each stage. Moreover, this study did not provide specific recommendations for the optimal intensity and duration in each exercise stage for Wistar rats with different ages. Only one duration (3 min in each stage) was observed in our design. Thus, changes in duration should be observed in further studies.

## Conclusion

Compared with the older Wistar rats, the young ones (4 and 8 weeks old) adapted to speed increase better in the VO_2max_ test. Hence, speed should be increased when designing the VO_2max_ test protocol for young Wistar rats. Conversely, under a moderate treadmill speed, older Wistar rats (10 and 16 months old) were more competent with increasing slope and more likely to reach the largest VO_2max_ value with lower stress responses and injury risks. The test program demonstrated that VO_2max_ declined with age in male Wistar rats. Finally, the relative exercise intensity (%VO_2max_) at different treadmill speeds and grades for male Wistar rats with different ages was quantified. Given the improvement in working economy with low oxygen consumption during the training period, %VO_2max_was only applied to untrained rats or subjects for the initial design intensity. Therefore, this study could be used as a reference for assessing aerobic capacity and designing exercise intensity in studies on exercise intervention with untrained male Wistar rats.

## Methods

### Experimental animals

Wistar rats aged 4 weeks (male, 174.64 ± 57.06 g, n = 40), 10 weeks (male, 372.30 ± 49.56 g, n = 40), 10 months (male, 616.74 ± 63.50 g, n = 40) and 16 months (male, 674.45 ± 36.39 g, n = 40) were housed separately, with a 12:12 h light–dark cycle, 23 °C ± 2 ℃ room temperature and 45%–55% humidity. Food and water were provided ad libitum. All animal procedures were performed in accordance with the experimental protocol approved by the Animal Ethical Committee of China Institute of Sports Science. After 1 week of adaptive feeding, all rats were trained to be familiar with the treadmill exercise program in 1 week. The maximum oxygen uptake was assessed with different test protocols.

### Test procedures

Twenty rats were selected randomly from each age group (4-week-old, 10-week-old and 10-month-old groups). Each rat completed three exercise protocols in random order within a 3-day interval. When all three exercise protocols were accomplished, we analysed VO_2max_, RER, completion time and induction rate (ratio of VO_2max_ induced cases to total tested rats’ cases) to determine an optimal test protocol of VO_2max_ for different age groups. In the second stage, we randomly selected another 20 rats from the four groups to test VO_2max_ using the preceding optimal test protocol. Accordingly, we explored the change in age-related VO_2max_ in Wistar rats. Finally, the relationship between relative exercise intensity (%VO_2max_) and treadmill speed and slope was quantified in the Wistar rats groups aged 4 weeks, 10 weeks and 16 months.

### Exercise protocol

**Figure 1:**
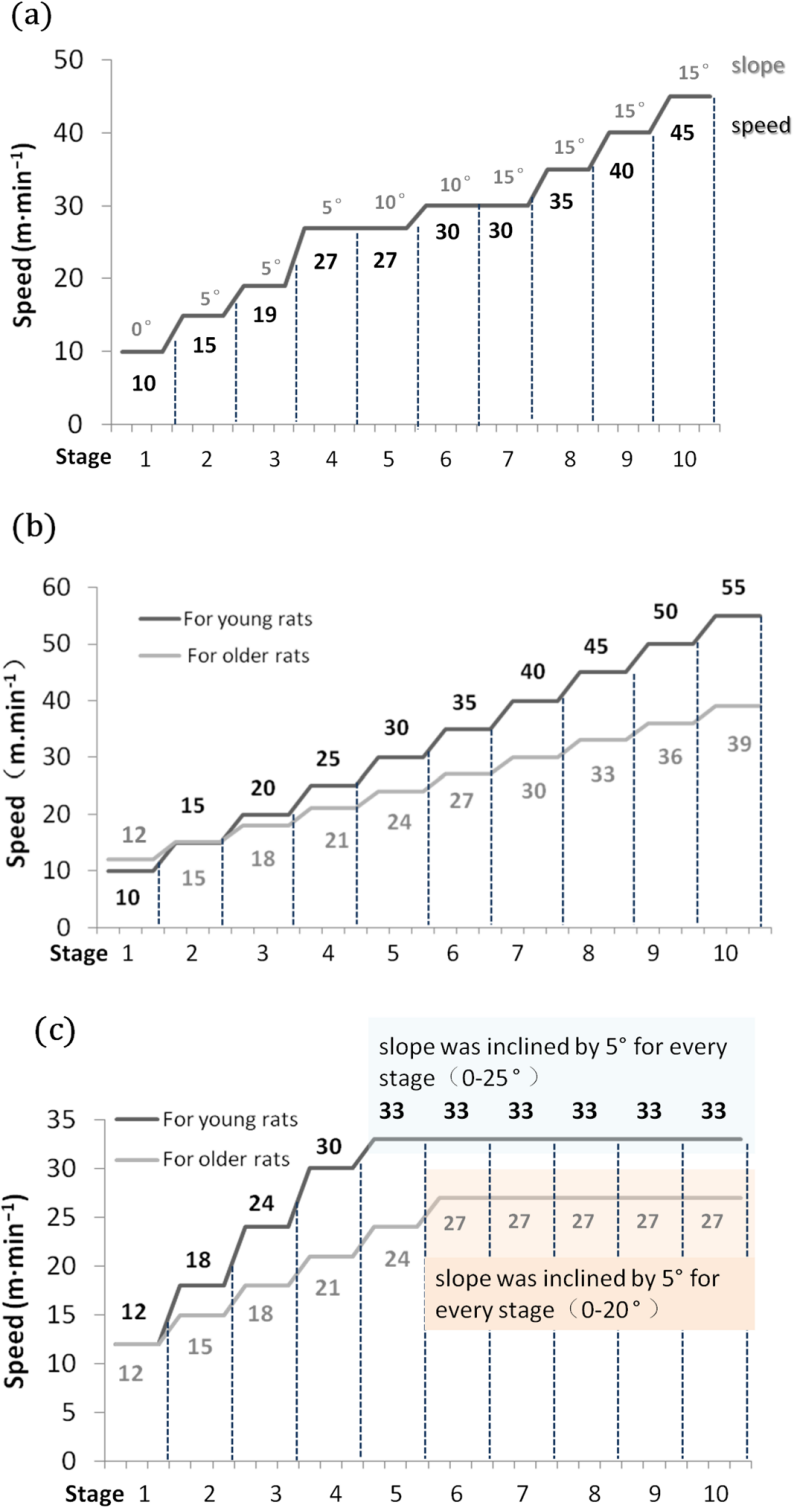
Treadmill testing procedure. (**a**) Treadmill testing procedure I. (**b**) Treadmill testing procedure II. (**c**) Treadmill testing procedure III. Each stage lasted for 3 min.

Procedure 1 (P1) involved a continuous and progressive increase in speed and slope, and it was designed by modifying the protocol from Bedford et al.^10^ (Fig. 1a). Procedure 2 (P2) was an incremental speed protocol with a 0° slope^12,13^. Treadmill speed was increased by 5 m min^−1^ every 3 min for young rats (4 week and 8 week age groups) or increased by 3 m min^−1^ for older rats (10 month age group) until the rats were exhausted (Fig. 1b). Procedure 3 (P3) was an incremental load protocol, in which speed was increased until approximately 80–85% of VO_2max_ was reached (the speed was determined according to previous pre-experimental results. Over this speed, the willingness to run in rats declined.). This speed was maintained, and the treadmill slope was inclined by 5° for every stage until the rats were exhausted (Fig. 1c). Treadmill speed was increased by 6 and 3 m min^−1^ every 3 min until 33 and 27 m min^−1^ were achieved by the young and older rats, respectively. Rats may produce a stress response when faced with an unfamiliar environment and unaccustomed behaviours, such as running on a treadmill. Therefore, 1-week adaptive treadmill running was performed to reduce stress and guarantee ideal results.

The exercise intensity (%VO_2max_) corresponding to different treadmill speeds was quantified at four grades (0°, 5°, 10° and 15°) for 4- and 8-week-old rats and at three grades (0°, 5° and 10°) for 16-month-old rats. Treadmill speed was increased by 5 and 3 m min^−1^ every 3 min for the young and older rats, respectively. All tests were separated by 3 days.

### Measurement of oxygen uptake

Experimental parameters, including resting metabolic rate (RMR), volume of oxygen uptake (VO_2_) and RER, were assessed using the Columbus Oxymax Lab Animal Monitoring System and an animal treadmill chamber. O_2_ and CO_2_ concentrations were measured as a percentage of the incoming continuous flow. A gas analyser was calibrated with ambient air and a reference gas (20.6% O_2_ and 0.501% CO_2_ + N_2_) in a room with stable temperature and humidity. The rats’ body masses were measured. Air was pumped through the treadmill chamber at a flow rate of 5.0 LPM. Samples of extracted air were detected by the oxygen and carbon dioxide analysers. We measured the RMRs after the rats had rested for 10 min in the test chamber to avoid possible external stress stimulus. The test protocols were performed, and data were collected every 30 s. Each rat had a 5 min warm up at 40–50% estimated VO_2max_ prior to the test. We used various criteria to confirm that VO_2max_ was elicited during the test. The criteria were as follows: the increase in VO_2_ was less than 5% with the increased workload, RER was above 1.00 and exhaustion characteristics (unwilling running, feeble kicking of hind legs and mental sluggishness) were observed^22,23^. Once any two of the above criteria were met, we stopped the test and recorded the VO_2max_ value.

### Statistical analysis

Measurement results were presented as mean ± standard deviation. Statistical analysis was performed using SPSS software (SPSS 22.0 for Windows, IBM SPSS Statistics). The assumptions of normality and variance homogeneity were tested. We conducted repeated measures to examine the differences in VO_2max_ indicators over the three procedures. One-way ANOVA was conducted to compare all measurements and multiple comparisons by least significant difference or Dunnett’s test among groups. A Kruskal–Wallis test was used for non-normally distributed data. The level of significance was set at *p* < 0.05.
